# Experiencing short heat waves early in development changes thermal responsiveness of turtle embryos to later heat waves

**DOI:** 10.1242/jeb.246235

**Published:** 2023-09-28

**Authors:** Anthony T. Breitenbach, Rosario A. Marroquín-Flores, Ryan T. Paitz, Rachel M. Bowden

**Affiliations:** ^1^ School of Biological Sciences, Illinois State University, Normal, IL 61790, USA; ^2^ Odum School of Ecology, University of Georgia, Athens, GA 30602, USA; ^3^ Department of Biological Sciences, Texas Tech University, Lubbock, TX 79409, USA

**Keywords:** Sex determination, Temperature fluctuation, Gene expression, Alternative splicing

## Abstract

Although physiological responses to the thermal environment are most frequently investigated using constant temperatures, the incorporation of thermal variability can allow for a more accurate prediction of how thermally sensitive species respond to a rapidly changing climate. In species with temperature-dependent sex determination (TSD), developmental responses to incubation temperature are mediated by several genes involved in gonadal differentiation. *Kdm6b* and *Dmrt1* respond to cool incubation temperatures and are associated with testis development, while *FoxL2* and *Cyp19A1* respond to warm incubation temperatures and are associated with ovary development. Using fluctuating incubation temperatures, we designed two studies, one investigating how conflicting thermal cues affect the timing of commitment to gonadal development, and another investigating the rapid molecular responses to conflicting thermal cues in the red-eared slider turtle (*Trachemys scripta*). Using gene expression as a proxy of timing of commitment to gonadal fate, results from the first study show that exposure to high amounts of conflicting thermal cues during development delays commitment to gonadal fate. Results from the second study show that *Kdm6b* splice variants exhibit differential responses to early heat wave exposure, but rapidly (within 2 days) recover to pre-exposure levels after the heat wave. Despite changes in the expression of *Kdm6b* splice variants, there was no effect on *Dmrt1* expression. Collectively, these findings demonstrate how short exposures to heat early in development can change how embryos respond to heat later in development.

## Teaser

**Summary:** Expression of genes involved in gonadal differentiation of turtle embryos in response to temperatures mimicking heat waves indicates conflicting thermal cues early in development affect responses to thermal cues later in development and thus sex determination.

## INTRODUCTION

As climate patterns change at an unprecedented pace (IPCC, 2018), research on how temperature affects biological processes is more important now than it has ever been. Studies that expose groups of individuals to different constant temperatures to see how these different temperatures affect phenotypic responses have revealed that traits such as fecundity (Huey et al., 1995; Ma et al., 2017), growth rate (Li and McClane, 2006) and time to flowering (Miller-Rushing and Inouye, 2009; Dorji et al., 2020) are all sensitive to changes in the thermal environment. For most of these thermosensitive traits, the phenotypic response is affected by both the temperature the organism is exposed to and the duration of their exposure to that temperature. For example, in the dark-eyed junco (*Junco hyemalis*), metabolic rate changes rapidly in response to cold temperatures (Bakken et al., 1991), but changes in body conductance occur only after prolonged exposure to cold temperatures (Stager et al., 2020). It is therefore important to understand not only how different temperatures influence phenotype but also how the duration of exposure affects phenotype. These issues become even more concerning in the face of climate change, as organisms are more likely to experience transient exposure to temperature extremes, such as heat waves, in the future (Stillman, 2019). As there is individual variation in responsiveness, when these transient exposures occur, it is essential to identify which organisms or individuals are responding to the short-term temperature exposure. While we expect that most, if not all, organisms will be affected by climate change, those with thermosensitive traits that are related to fitness are likely to suffer the greatest consequences.

Species with temperature-dependent sex determination (TSD) may be particularly vulnerable to changing temperatures as population sex ratios are strongly affected by the thermal environment (Laloë et al., 2014; Jensen et al., 2018). TSD is widespread in reptiles (Ewert et al., 1994; Lang and Andrews, 1994; Harlow and Shine, 1999; Deeming, 2004; Shine et al., 2007), with thermal thresholds for the conditions that produce males and females. These thresholds were characterized by placing eggs at different constant temperatures for the entirety of development and quantifying resulting sex ratios (Bull and Vogt, 1979, 1981; Bull et al., 1982; Wibbels et al., 1998; Ewert et al., 1994). In the type of TSD that is most common in turtles, such as the red-eared slider (*Trachemys scripta*), cool constant temperatures (26°C) during embryonic development produce mainly males while warm constant temperatures (31°C) produce mainly females (Ewert and Nelson, 1991). Between these two temperatures is the pivotal temperature (∼29°C), the constant temperature that, when applied for the duration of embryonic development, produces a population-wide 50:50 sex ratio (Mrosovsky and Pieau, 1991; Godfrey and Mrosovsky, 2006). Thus, temperatures below the pivotal temperature are commonly referred to as male-producing temperatures (MPTs) while temperatures above the pivotal temperature are commonly referred to as female-producing temperatures (FPTs). Initial investigations into temporal thresholds shifted eggs to different constant temperatures (eggs typically shifted once or twice during development) to identify the stages of development where temperatures were capable of influencing sex ratios (Wibbels et al., 1991; Valenzuela, 2001; Pewphong et al., 2020). This work demonstrated that, under constant temperatures, the effects of incubation temperature are primarily limited to the middle third of embryonic development, the thermosensitive period (TSP; Wibbels et al., 1991), embryonic stages 15–20 (Greenbaum, 2002), and that incubation temperatures prior to or after this period do not affect resulting sex ratios. Incubation temperatures experienced after the TSP are thought to occur after the gonads have become committed to developing as either testes or ovaries, and thus come too late to affect sex ratios (Wibbels et al., 1991). Therefore, to understand how temperature affects sex ratios, research needs to focus on the period of development prior to when gonadal fate is committed. Of course, laboratory incubations using constant temperatures are not reflective of the thermal variability that organisms experience under natural conditions and may skew the timing of when gonadal fate becomes committed (Breitenbach et al., 2022), as prior modeling has suggested that the timing of the TSP varies under natural, more variable thermal conditions (Georges et al., 2005; Girondot et al., 2018).

More recent research has begun to bridge the gap between laboratory and natural conditions by investigating how exposure to more variable temperatures affects sex ratios (Georges et al., 1994; Du et al., 2009; Bowden et al., 2014; Carter et al., 2018, 2019; Valenzuela et al., 2019). Research using fluctuating temperatures and simulated heat waves demonstrates that in *T. scripta*, even short bouts of exposure (5 days) to warm temperatures are sufficient to produce some female hatchlings, with ∼8 days of exposure to warm temperatures producing a 50:50 sex ratio (Carter et al., 2018). However, some embryos developed testes even when experiencing up to 15 days of exposure to warm temperature (Carter et al., 2018). Furthermore, the timing and continuity of heat exposure also affects sex ratios, as heat waves applied in the middle of development produced female-biased sex ratios, while heat waves applied earlier or later than this window did not produce female-biased sex ratios (Breitenbach et al., 2020). Compared with continuous heat wave controls, a 1 day gap between heat waves, when temperatures drop back to cool conditions, does not significantly reduce the proportion of females produced, but a gap of 3 or more days dramatically reduces the proportion of resulting female hatchlings despite there being no difference in the overall number of heat wave days (Breitenbach et al., 2020). These findings demonstrate that embryos integrate incubation temperatures to regulate gonadal development until a point where they commit to testis or ovary development, and after this point incubation temperatures no longer affect gonadal fate. There appears to be substantial individual variation in how long it takes an embryo to reach the point of commitment to gonadal fate and this variation may be critical to understanding how transient temperature exposures affect sex ratios under natural incubation conditions.

**Fig. 1.**
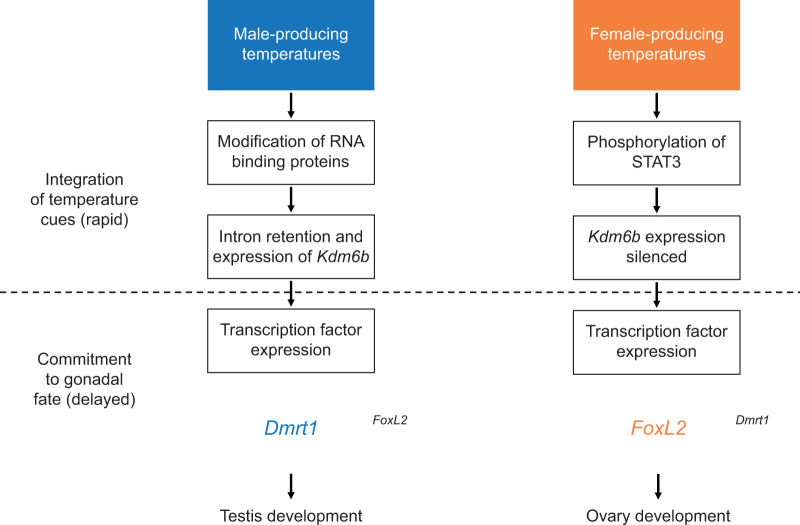
Rapid and delayed responses under male- and female-producing temperatures that ultimately lead to testis and ovary development, respectively.

Recent research has shed light on the molecular events that transduce incubation temperature into biochemical events that drive the expression of genes involved in gonadal differentiation in vertebrates with TSD (Czerwinski et al., 2016; Deveson et al., 2017; Bock et al., 2020), including *T. scripta* (Ge et al., 2018; Weber et al., 2020; Marroquín-Flores et al., 2021)*.* Many of the genes involved in gonadal development in species with TSD are also involved in gonadal differentiation in species with genetic sex determination (GSD) (Smith et al., 1999; Loffler et al., 2003; Ferguson-Smith, 2007; Capel, 2017). In a broad sense, TSD appears to result from temperature-sensitive protein function modulating the epigenetic regulation of genes involved in gonadal differentiation. While the complete sequence of events is not fully resolved, some of the events have been characterized (Fig. 1). At male-producing temperatures, a histone demethylase that exhibits temperature-sensitive intron retention, *Kdm6b*, is upregulated (Deveson et al., 2017; Marroquín-Flores et al., 2021), and this intron retention appears to be regulated by temperature-sensitive phosphorylation of RNA binding proteins (Haltenhof et al., 2020). *Kdm6b* subsequently induces the expression of *Dmrt1*, a highly conserved transcription factor involved in testis development for most vertebrates, by demethylating its promoter (Ge et al., 2018), and this is hypothesized to drive testis development. When eggs are incubated at FPTs, signal transducer and activator of transcription 3 (STAT3) is phosphorylated in response to Ca^2+^ influx and subsequently silences *Kdm6b* expression (Weber et al., 2020). Additionally, under FPTs, the expression of *FoxL2* is induced and this codes for a transcription factor that upregulates other genes involved in ovary development, such as *Cyp19A1* (Matsumoto and Crews, 2012; Matsumoto et al., 2013). *Cyp19A1* codes for aromatase, which catalyzes the conversion of androgens into estrogens and, when present, increases the amount of estrogens in the embryonic gonads (Lance, 2009). Similar to *Dmrt1* under MPTs, *FoxL2* and *Cyp19A1* also exhibit delayed responses to sex-specific incubation cues compared with *Kdm6b* (Breitenbach et al., 2020; Marroquín-Flores et al., 2021). As our understanding of the cascade of events that lead from temperature cues to changes in gonadal development advances, there appear to be rapid changes in response to temperature, such as *Kdm6b* expression, that may represent more general temperature responses that could affect a suite of the well-established temperature-sensitive processes in reptiles, one of which would be gonad differentiation. The process of gonadal differentiation, in turn, may be responding to indirect thermal cues such as histone demethylation mediated by *Kdm6b* rather than directly responding to temperature itself, as evidenced by many downstream genes differing in how long they take to respond to temperature cues.

We are just now beginning to understand how the expression of these genes changes in response to transient temperature exposures, and the findings demonstrate that there is substantial variation in how long it takes different genes to respond to temperature. In response to exposure to warm temperatures, *Kdm6b* expression significantly decreases within hours (Bock et al., 2020) to days (Marroquín-Flores et al., 2021), while *Cyp19A1* expression can take days to weeks to increase (Breitenbach et al., 2020). This may be due to the different functions of these genes and the proteins they encode; *Kdm6b* is involved in integrating temperature cues and *Cyp19A1* expression increases after commitment to ovary development (Fig. 1). We do not know which genes, if any, show a change in expression indicative that gonadal fate has become committed, but as *Kdm6b* expression changes just hours after a temperature change (Bock et al., 2020), a change in the expression of *Kdm6b* is not indicative of gonadal commitment. Using *T. scripta* as an example, a 2 day heat wave reduces *Kdm6b* expression (Marroquín-Flores et al., 2021), but it is not sufficient to induce ovary development (Carter et al., 2018). By studying how different genes respond to variable incubation conditions, it may become possible to identify genes that are associated with the commitment to a particular gonadal fate and use these genes to study variation in how individuals respond to incubation temperatures. Variation in this ‘thermal responsiveness’ (Bowden and Paitz, 2018) could be critical to adapting to changing climates that involve transient exposures to unseasonal temperatures.

In the current study, we characterized how embryos respond to transient exposure to both MPT and FPT cues, thereby mimicking the thermal conditions embryos would be exposed to during development. We designed two studies to investigate how transient temperature exposure affects the expression of genes on different time scales. First, we hypothesized that embryos delay the commitment of gonadal fate in response to conflicting thermal cues. To test this hypothesis, we exposed *T. scripta* embryos to five different incubation conditions varying in the degree of conflicting thermal cues and sampled gonads across embryonic development. We analyzed the expression of two genes involved in testis development (*Kdm6b* and *Dmrt1*) and two genes involved in ovary development (*FoxL2* and *Cyp19A1*) and used the resulting expression patterns as an indicator of the trajectory of gonadal differentiation into either testes or ovaries in response to our different incubation treatments. Second, we hypothesized that short exposures to heat waves would not be sufficient to silence the male pathway. To test this hypothesis, we quantified the abundance of *Kdm6b* transcripts, with (+IR) and without (−IR) a retained intron, before and after exposure to a fluctuating FPT for 2 days. We also analyzed *Dmrt1* expression after heat exposure to capture downstream changes in gene expression.

## MATERIALS AND METHODS

### Study 1: delayed responses to conflicting thermal cues

**Fig. 2.**
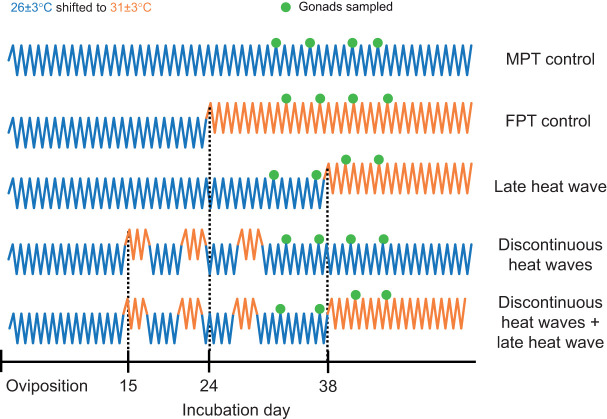
**Incubation treatments with varying degrees of conflicting thermal cues from the delayed response study (study 1).** At each sampling point, both gonads were sampled from 4–7 individuals. MPT, male-producing temperature; FPT, female-producing temperature.

*Trachemys scripta* (Thunberg and Schoepff 1792) eggs were shipped from Concordia Turtle Farm, LLC (Jonesville, LA, USA) to the laboratory at Illinois State University. Eggs were placed in moist vermiculite maintained at −150 kPa and randomly assigned to 1 of 5 incubation treatments (Fig. 2); while this study did not examine sex ratios, we have prior sex ratio data on 4 of the 5 treatments used (Breitenbach et al., 2020). In the first treatment (MPT control), eggs experienced daily temperature fluctuations of 26±3°C for the entirety of development and this produces males. In the second treatment (FPT control), eggs experienced daily temperature fluctuations of 26±3°C until day 24 of development, when they were shifted to daily fluctuations of 31±3°C and this produces females. In the third treatment (late heat wave), eggs experienced a shift from 26±3°C to 31±3°C on day 38 of development and this produces a male-biased sex ratio, likely because the heat exposure occurs after commitment to testis development. In the fourth treatment (discontinuous heat waves), eggs experienced early heat exposure with three separate 3 day bouts at 31±3°C while the rest of development was spent at 26±3°C and this also produces a male-biased sex ratio. The fifth treatment (discontinuous heat waves+late heat wave) was a combination of treatments three and four where eggs experienced the three discontinuous heat waves as well as the late shift to 31±3°C on day 38 of development. We do not have a predicted sex ratio outcome for this treatment. As neither discontinuous heat waves nor late heat waves induce ovary development, it is possible that embryos experiencing both conditions still may not experience a cue for ovary development. Conversely, experiencing both conditions may be sufficient to induce ovary development even though embryos will be at even more advanced stages of development when they experience the late heat exposure as a result of early heat exposure increasing developmental rates.

Eggs were shifted between incubators programmed to run a cool, male-producing daily fluctuation of 26±3°C (IPP 110 Plus, Memmert GmbH+Co. KG, Schwabach, Germany) and incubators programmed to run a warm, female-producing daily fluctuation of 31±3°C (IPP 400, Memmert GmbH+Co. KG) depending upon the incubation treatment. Egg containers were shifted regularly between different positions inside incubators to minimize position effects within the incubator. No eggs were allowed to hatch in this study.

In all treatments, embryonic gonads were sampled at four different stages during embryonic development. Sampling points were designed to allow gene expression analysis over the thermosensitive period (Breitenbach et al., 2020). Gene expression was quantified using 4–7 sampled individuals per treatment per sampling point; both embryonic gonads were collected from each sampled individual. During each sampling point, gonads were immediately placed in TRIzol reagent (Ambion) and stored at −80°C until RNA extraction via 2-propanol (Fisher Chemical) and chloroform (Thermo Scientific). cDNA was then synthesized from 1 mg of RNA using the Maxima First Strand cDNA Synthesis Kit for RT-qPCR with dsDNase (Thermo Scientific) based upon the manufacturer's protocol. qPCR was performed using triplicates of each sample and PowerUp SYBR Green Master Mix (Applied Biosystems). Published primers were used for *Cyp19A1* (Ramsey et al., 2007), *Kdm6b* (Marroquín-Flores et al., 2021), *Dmrt1* (Shoemaker et al., 2007) and *Gapdh*, which was used as a housekeeping gene (Breitenbach et al., 2020, 2022). The primers for *Kdm6b* specifically targeted the intron-retaining splice variant (+IR). Primers for *FoxL2* were designed using the *T. scripta* genome (Simison et al., 2020), and amplified sequence identity was confirmed by sending PCR products to the Core/Sanger Facility (Roy J. Carver Biotechnology Center, University of Illinois at Urbana-Champaign). Relative expression was calculated using the ΔΔCT method (Rao et al., 2013; Breitenbach et al., 2020, 2022).

All statistical analyses were performed in R (http://www.R-project.org/). Gene expression data were analyzed using separate generalized linear models (GLMs) for each analyzed gene, using temperature treatment and sampling day range as fixed effects. The GLM distribution that best fitted model assumptions was specified for each gene, and data were transformed to meet model assumptions. Estimated marginal means (R package: https://CRAN.R-project.org/package=emmeans) were used for *post hoc* comparisons. All *post hoc* comparisons, which used false discovery rate (FDR) adjustments to control for the probability of type I error, were used to compare expression levels across treatments within the last sampling day range only, as the latest (developmentally) sampling point is most indicative of the trajectory of gonadal development.

### Study 2: rapid responses to conflicting thermal cues

**Fig. 3.**
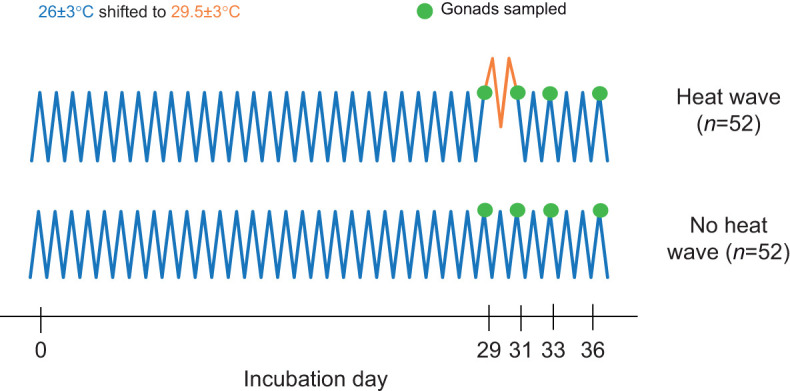
**Incubation treatments from the rapid response study (study 2).** At each sampling point, both gonads were sampled from 4–8 individuals.

A concurrent study was conducted to investigate more rapid changes in gene expression in response to temperature. *Trachemys scripta* eggs were shipped from Concordia Turtle Farm LLC and processed as described above. Upon arrival, eggs were randomly sorted into two treatment groups (Fig. 3). Eggs assigned to the control treatment were incubated (IPP 110 Plus, Memmert GmbH+Co. KG) at a fluctuating MPT of 25.0±3°C. Eggs assigned to the heat wave treatment were incubated at MPT for the first 29 days of incubation (IPP 400, Memmert GmbH+Co. KG) before being shifted to a fluctuating FPT of 29.5±3°C for just 2 days. Post-heat wave, eggs were returned to MPT until incubation day 36. While the fluctuating regimes used for study 2 differ slightly from those used in study 1, fluctuating MPT regimes ranging from 25.0±3°C to 27.0±3°C produce 100% male hatchlings (Carter et al., 2018; Breitenbach et al., 2020). Similarly, fluctuating FPT regimes (±3°C) with a mean above 29.5°C should induce 100% female hatchlings (Carter et al., 2018; Breitenbach et al., 2020). The fluctuating regimes used in study 2 were selected to mimic incubation conditions from Marroquín-Flores et al. (2021) as a direct follow-up to that study. No eggs were allowed to hatch in this study.

Gonads were dissected from 4–8 individuals in both treatments on days 29, 31, 33 and 36 to capture changes in gene expression before and after the heat wave. Gonadal tissues were placed in 1 ml of Trizol. RNA from embryonic gonads was extracted using 2-propanol (Fisher Chemical) and chloroform (Thermo Scientific). cDNA was synthesized using a Maxima First Strand cDNA Synthesis Kit (Thermo Scientific), following the manufacturer's protocol, and samples were standardized to a concentration of 62.5 ng ml^−1^. qPCR was used to capture changes in the expression of *Kdm6b* and *Dmrt1*, using PowerUp SYBR Green Master Mix (Applied Biosystems). Three primers were used to characterize alternative splicing in *Kdm6b*. *Kdm6b*(+IR) was used to measure intron retention and *Kdm6b*(−IR) was used to measure the expression of the transcript lacking the retained intron (Marroquín-Flores et al., 2021). The final set of primers, *Kdm6b*Ge, was used to capture non-discriminate *Kdm6b* expression (Ge et al., 2018). *Gapdh* was used as a housekeeping gene to normalize gene expression (Breitenbach et al., 2020). The ΔΔCT method (Rao et al., 2013; Breitenbach et al., 2020) was used to calculate relative gene expression.

All statistical analyses were performed in R (http://www.R-project.org/). A GLM was used to analyze changes in gene expression between the two treatment groups. All data were log transformed to meet the assumptions of the model. Treatment and incubation day were used as fixed effects and estimated marginal means (R package: https://CRAN.R-project.org/package=emmeans) were used for *post hoc* comparisons between treatments within and between sampling days. FDR adjustment was used to adjust for multiple tests in *post hoc* comparisons.

## RESULTS

### Study 1: delayed responses to conflicting thermal cues

**Fig. 4.**
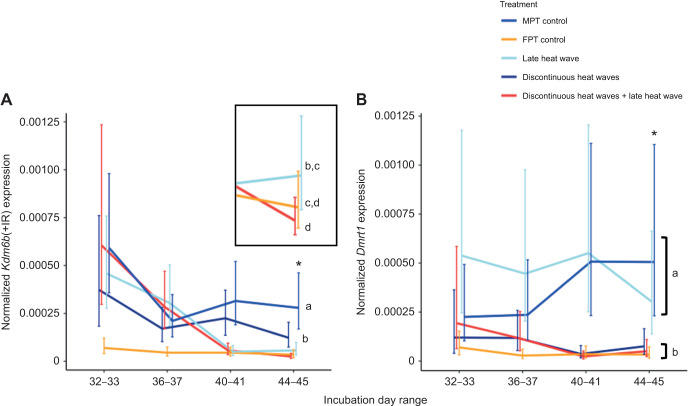
**Resulting gene expression patterns of genes involved in testis development (study 1).** (A) *Kdm6b*(+IR) and (B) *Dmrt1* expression. Incubation treatments are as described in Fig. 2. Expression was normalized to that of *Gapdh* and is expressed as the mean±95% confidence interval (CI). Asterisks denote significant differences between treatments in only the last incubation day range; for each gene, treatments that do not share a letter are significantly different in expression. *Post hoc* comparisons were done between treatments in the last sampling day range only.

**Fig. 5.**
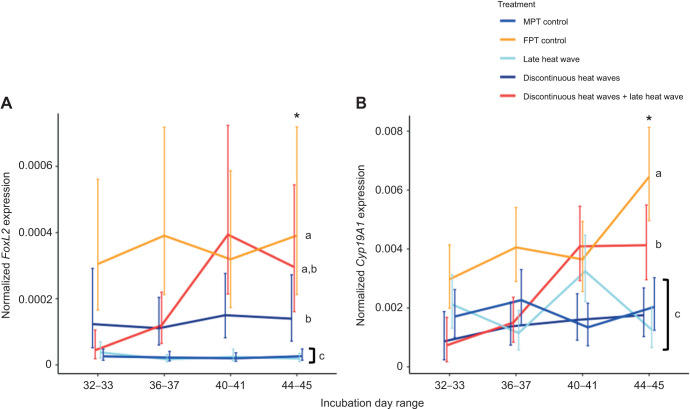
**Resulting gene expression patterns of genes involved in ovary development (study 1).** (A) *FoxL2* and (B) *Cyp19A1* expression. Incubation treatments are as described in Fig. 2. Expression was normalized to that of *Gapdh* and is expressed as the mean±95% CI. Asterisks denote significant differences between treatments in only the last incubation day range; for each gene, treatments that do not share a letter are significantly different in expression. *Post hoc* comparisons were done between treatments in the last sampling day range only.

There was a significant interaction between temperature treatment and sampling day for every gene we quantified. *Kdm6b*(+IR) (χ^2^=66.79, d.f.=12, *P*<0.0001), *Dmrt1* (χ^2^=22.64, d.f.=12, *P*=0.03), *FoxL2* (χ^2^=23.43, d.f.=12, *P*=0.02) and *Cyp19A1* (χ^2^=47.56, d.f.=12, *P*<0.0001) were all affected by incubation treatments, but this effect varied across sampling days. Gene expression trends for each treatment are shown in Fig. 4 [*Kdm6b*(+IR) and *Dmrt1*] and Fig. 5 (*FoxL2* and *Cyp19A1*), and results of *post hoc* tests between treatments on the last sampling day range are shown in the figures.

In general, the MPT control treatment and the late heat wave treatment exhibited higher expression of testis-associated genes by the last sampling point. For the MPT control treatment, expression of *Kdm6b*(+IR) and *Dmrt1* was significantly higher than that for all other treatments except that *Dmrt1* expression was similar to that for the late heat wave treatment. The late heat wave treatment also exhibited higher *Kdm6b*(+IR) and *Dmrt1* expression than all other treatment groups except the MPT control treatment.

In general, the FPT control treatment and the discontinuous heat waves+late heat wave treatment exhibited higher expression of ovary-associated genes by the last sampling point. For the FPT control treatment, *Cyp19A1* expression was significantly higher than that of all the other treatments, and *FoxL2* expression was higher than that of all the other treatment groups except the discontinuous heat waves+late heat wave treatment. The discontinuous heat waves+late heat wave treatment also exhibited elevated *FoxL2* and *Cyp19A1*. In this treatment, *FoxL2* expression was similar to that in the FPT control treatment and the discontinuous heat wave treatment, but higher than that in the MPT control treatment and the late heat wave treatment. *Cyp19A1* expression was not as high as for the FPT control treatment, but still higher than that for the other three treatments.

For the discontinuous heat wave treatment, there was no apparent induction of testis or ovary gene expression. *Post hoc* tests showed that *Dmrt1* expression was significantly lower than for the MPT control treatment and comparable to that for the FPT control treatment. Expression of *FoxL2* was intermediate, being significantly lower than that for the FPT control treatment and significantly higher than that for the MPT control treatment. *Cyp19A1* expression was also low and comparable to that for the MPT control treatment and significantly lower than that for the FPT control treatment. *Kdm6b*(+IR) expression was intermediate, being significantly lower than that for the MPT control treatment and significantly higher than that for the FPT control treatment.

### Study 2: analysis of rapid responses of genes in the male pathway

**Fig. 6.**
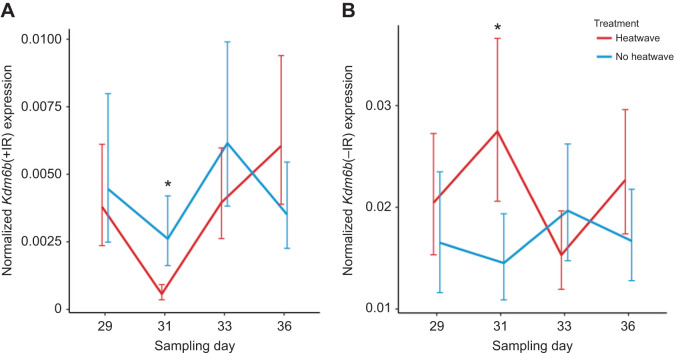
**Resulting expression of the alternatively spliced transcripts (study 2).** (A) *Kdm6b*(+IR) and (B) *Kdm6b*(−IR) expression. Incubation treatments are as described in Fig. 3. Expression was normalized to that of *Gapdh* and is expressed as the mean±95% CI. Asterisks denote significant differences between treatments within a sampling day.

**Fig. 7.**
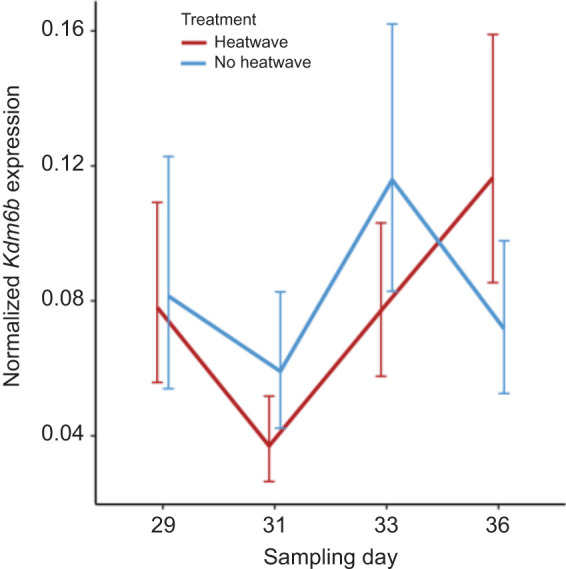
**Resulting overall expression of *Kdm6b* (study 2).** Incubation treatments are as described in Fig. 3. Expression of *Kdm6b*Ge was normalized to that of *Gapdh* and is expressed as the mean±95% CI. There were no significant differences between treatments within sampling days.

**Fig. 8.**
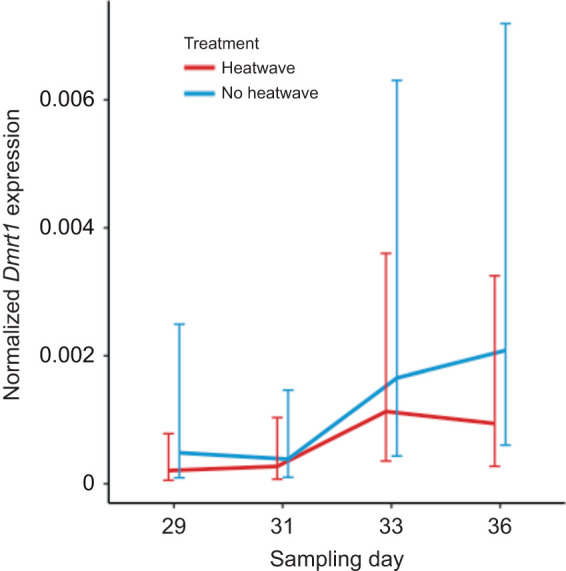
**Resulting *Dmrt1* expression (study 2).** Incubation treatments are as described in Fig. 3. Expression of *Dmrt1* was normalized to that of *Gapdh* and is expressed as the mean±95% CI. There were no significant differences between treatments within sampling days.

When quantifying *Kdm6b*(+IR), we found a significant interaction effect between treatment and incubation day (χ^2^=21.129, d.f.=3, *P*<0.001; Fig. 6A), where expression was significantly lower while the eggs were experiencing the heat wave on incubation day 31 (*P*<0.001), but quickly returned to similar levels to the baseline group just 2 days after the heat wave (*P*=0.1649). When quantifying *Kdm6b*(−IR), we also identified a significant interaction effect (χ^2^=10.7160, d.f.=3, *P*<0.05; Fig. 6B), where *Kdm6b*(−IR) expression was essentially the opposite of *Kdm6b*(+IR) expression. Here, levels of *Kdm6b*(−IR) increased on incubation day 31 in the heat wave treatment group (*P*<0.01), but quickly returned to similar levels to the baseline group just 2 days after the heat wave (*P*=0.1916). The global test on non-discriminate *Kdm6b* expression identified a significant interaction effect between treatment and incubation day (χ^2^=11.6893, d.f.=3, *P*<0.01; Fig. 7), but none of the pairwise tests of differences on the interaction effects were statistically significant after controlling for multiple testing. Similarly, the global test on *Dmrt1* expression identified a significant effect of incubation day (χ^2^=0.2434, d.f.=3, *P*<0.05; Fig. 8), but none of the pairwise tests of differences between days were statistically significant after controlling for multiple testing.

## DISCUSSION

The reality for many organisms is that the temperatures they experience are routinely changing and to regulate physiological processes, a range of thermal cues must be properly integrated. In the current study, we set out to investigate how embryos respond to transient exposure to temperatures during development. In our first study, we characterized gene expression under incubation conditions that varied in the amount of conflicting thermal cues the embryos experienced. Based on our treatments, embryos experienced low (MPT control, FPT control or late heat wave), moderate (discontinuous heat wave) or high (discontinuous heat waves+late heat wave) amounts of conflicting thermal cues during development. Embryos in the control groups exhibited gene expression patterns consistent with the expected gonadal fate, with embryos in the MPT control treatment having elevated *Kdm6b* and *Dmrt1* expression and embryos in the FPT control treatment having elevated *FoxL2* and *Cyp19A1* expression. Gene expression patterns in the late heat wave group also occurred as expected as testis-associated genes were elevated, and the late heat wave did not reduce this expression. This is consistent with the finding that sex ratios under these conditions are male biased (Breitenbach et al., 2020) because heat exposure is thought to happen after embryos have committed to testis development.

One of our primary goals was to examine how short exposures to heat early in development might affect the timing of when embryos commit to testis development. Embryos in the discontinuous heat wave treatment experienced three bouts of 3 day heat waves and we found that exposure to these heat waves changed gene expression in a manner that appeared to delay when embryos commit to testis development. Embryos in the discontinuous heat wave treatment were the only embryos that did not show elevated *Dmrt1* expression or *Cyp19A1* expression, in that *Dmrt1* expression was similar to that of the FPT control treatment embryos and *Cyp19A1* expression was similar to that of the MPT control treatment embryos (Figs 4B and 5B). Our interpretation of this pattern is that embryos had yet to commit to a gonadal fate by this stage of development. Previous studies suggest embryos developing under these conditions would hatch as males (Breitenbach et al., 2020), but it is clear that *Dmrt1* expression was not induced by the last sampling point. This delay in the induction of gene expression appears to have functional consequences for the embryo by prolonging the time during which they are capable of responding to heat waves. Embryos in the discontinuous heat waves+late heat wave treatment responded to the late heat wave despite the fact they were at a more advanced developmental stage than the embryos in the late heat wave group that did not respond to the late heat wave. Conflicting thermal cues early in development can thus affect how embryos respond to thermal cues later in development and alter the effect of these late cues on sex determination.

Our second study characterized gene expression during early heat wave exposure to examine the rapid response to temperature cues. Embryos exposed to just 2 days of heat experienced a decrease in the expression of *Kdm6b*(+IR), coupled with an increase in the expression of *Kdm6b*(−IR), which is consistent with temperature-sensitive splicing of the intron (Haltenhof et al., 2020). When heat exposure subsided for 2 days, both *Kdm6b*(+IR) and *Kdm6b*(−IR) expression returned to levels comparable to those of embryos that did not experience heat exposure. *Kdm6b* expression did not respond to this early heat wave exposure when it was measured using primers that could not differentiate between the *Kdm6b* splice variants. These findings are consistent with prior work suggesting that *Kdm6b* splice variants respond rapidly to changes in incubation temperature and that these changes occur prior to changes in overall *Kdm6b* expression (Marroquín-Flores et al., 2021). As expected, *Dmrt1* did not respond to early heat wave exposure despite the changes in *Kdm6b*. *Dmrt1* is a gene that exhibits delayed expression under fluctuating incubation conditions, relative to constant conditions (Shoemaker-Daly et al., 2010; Ge et al., 2018; Breitenbach et al., 2020; this study). Prior work using fluctuating temperatures suggests that increases in *Kdm6b*(+IR) and overall *Kdm6b* expression occur several developmental stages prior to the induction of *Dmrt1* (Marroquín-Flores et al., 2021). Our data capture the initial cascade of events, which take place before we see changes in downstream gene expression and the commitment to male or female development. Under natural conditions, the rapid response of genes such as *Kdm6b*(+IR) may integrate conflicting thermal cues which accumulate to induce downstream gene expression and commitment to gonadal fate.

By examining changes in gene expression on different time scales, we were able to demonstrate that the expression of some genes can respond rapidly to changing temperatures (hours to days), while other downstream genes have a much longer response time to changing temperatures (days to weeks). Only through the use of more naturalistic incubation conditions are we able to characterize variation in how organisms respond to transient temperature exposures and what the functional consequences of transient exposures are for the organism. These results are important in the context of a changing climate, as heat waves are predicted to increase in frequency, intensity and length (Hayhoe et al., 2010; Rahmstorf and Coumou, 2011; Stillman, 2019) and advance our understanding of how changes in gene expression underlie the integration of temperature cues to drive a temperature-dependent phenotype.

While our findings have important implications for understanding how TSD operates under more natural conditions, the finding that exposure to conflicting thermal cues can potentially delay biological responses also has broader implications for multiple thermally sensitive traits in other taxa. Exposure to conflicting thermal cues may delay when individuals migrate. This can affect fitness and/or survival, as delayed migration can decrease reproductive fitness in birds (Baker et al., 2004; Gienapp and Bregnballe, 2012) and survival in fish (Minke-Martin et al., 2018; Jensen et al., 2022). Delayed hibernation emergence in response to temperature cues can also negatively affect fitness, as has been demonstrated in Columbian ground squirrels (Lane et al., 2012). Delayed metamorphosis may also decrease growth rate, as has been shown in barnacles (Pechenik et al., 1993). While laboratory studies using controlled temperatures that are more ecologically relevant than constant temperatures, like those reported here, can shed light on how specific attributes of the thermal environment (e.g. conflicting thermal cues) can affect thermally sensitive traits, future studies should investigate whether these patterns remain true under natural conditions. Ultimately, as studies using constant temperatures in the laboratory likely mask the individual variation in when responses manifest, it is even more critical to design studies with ecological relevance in mind, especially in the face of a rapidly changing climate.

### Conclusions

We sought to investigate the delayed and rapid responses to more variable incubation temperatures in a turtle with TSD. From a delayed response perspective, the resulting expression patterns of genes involved in gonadal development [*Kdm6b*(+IR), *Dmrt1*, *FoxL2*, *Cyp19A1*] suggest that exposure to conflicting thermal cues appears to delay commitment to gonadal fate to stages outside of those historically thought of as the thermosensitive period (stages 21–22). Whether there is a functional consequence to this delay in commitment to gonadal fate remains to be determined. From a rapid response perspective, our findings suggest that intron retention may enable incubating embryos to sense and respond to changing environmental temperatures. Prolonged heat exposure leads to downregulation of *Kdm6b*, which may be driven by cumulative splicing of the *Kdm6b* transcript after several days. While *Kdm6b*(+IR) can recover quickly, splicing of the *Kdm6b* transcript may contribute to the heat sensitivity of embryos, which makes them subject to the effects of heat waves. Species with TSD will continue to face the effects of climate warming events and these data contribute to our understanding of how embryos will respond to heat exposure during development. Collectively, these findings demonstrate the importance of utilizing more naturalistic thermal conditions in laboratory studies on multiple time scales.
